# Adams-Oliver Syndrome in a Newborn: A Case Report and Comprehensive Literature Review

**DOI:** 10.7759/cureus.86442

**Published:** 2025-06-20

**Authors:** Chaimae N'joumi, Anass Ayyad, Sahar Messaoudi, Rim Amrani

**Affiliations:** 1 Department of Pediatrics, Centre Hospitalier Universitaire, Oujda, MAR; 2 Department of Neonatology and Neonatal Resuscitation, Faculty of Medicine and Pharmacy of Oujda, Mother and Child Health Laboratory, Oujda, MAR

**Keywords:** adams–oliver syndrome, aplasia cutis congenita, brachydactyly, rare genetic syndrome, terminal limb defects

## Abstract

Adams-Oliver syndrome (AOS) is a congenital condition marked by aplasia cutis congenita and terminal limb defects, often accompanied by diverse systemic manifestations. We present a case of a newborn female with scalp aplasia cutis and brachydactyly, but no internal organ involvement. Clinical and imaging assessments identified a scalp bone defect without neurological or cardiac abnormalities. Surgical correction of the scalp lesion was successfully performed. This case underscores the variable clinical presentation of AOS and highlights the need for comprehensive evaluation and multidisciplinary care. Early diagnosis and genetic counseling remain crucial for accurate prognosis and informed family planning.

## Introduction

Adams-Oliver syndrome (AOS) is a rare, clinically heterogeneous congenital disorder first described by Adams and Oliver in 1945 [1]. It is classically defined by the co-occurrence of aplasia cutis congenita (ACC), typically affecting the scalp along the parietal or occipital midline, and terminal transverse limb anomalies, which most commonly involve hypoplasia or aplasia of the distal phalanges [1]. In more severe phenotypes, limb reduction defects may extend to complete absence of digits, hands, or lower extremities, with occasional involvement of the metacarpals or metatarsals [2].

In addition to its cardinal features, AOS may encompass a broad spectrum of systemic anomalies, underscoring its polymalformative nature. Notably, structural abnormalities involving the cardiovascular system, central nervous system, and gastrointestinal tract have been documented, often correlating with poor clinical outcomes [3].

The wide phenotypic spectrum of AOS, from isolated anomalies to severe multisystem involvement, requires heightened clinical vigilance and a multidisciplinary diagnostic approach. Early identification is essential for guiding management, informing prognosis, and providing accurate genetic counseling and risk assessment for families.

We report a case of a newborn presenting the hallmark features of AOS, likely sporadic, with a mild phenotype focused on cutaneous scalp defects and no associated visceral complications.

## Case presentation

We present a case of a female neonate, the first child of a non-consanguineous couple, born at 38 weeks of gestation via spontaneous vaginal delivery, following an uneventful pregnancy. At birth, she weighed 3000 g, had Apgar scores of 10 at both one and five minutes, and a cephalic perimeter measuring 34 cm. The 34-year-old primigravid mother and the 45-year-old father denied any familial history of AOS, neurodevelopmental disorders, or congenital anomalies. The antenatal period was unremarkable, with no reported exposure to infections, radiation, or teratogenic substances such as medications, tobacco, alcohol, or recreational drugs. There was no history of perinatal trauma or complications during delivery.

**Figure 1:**
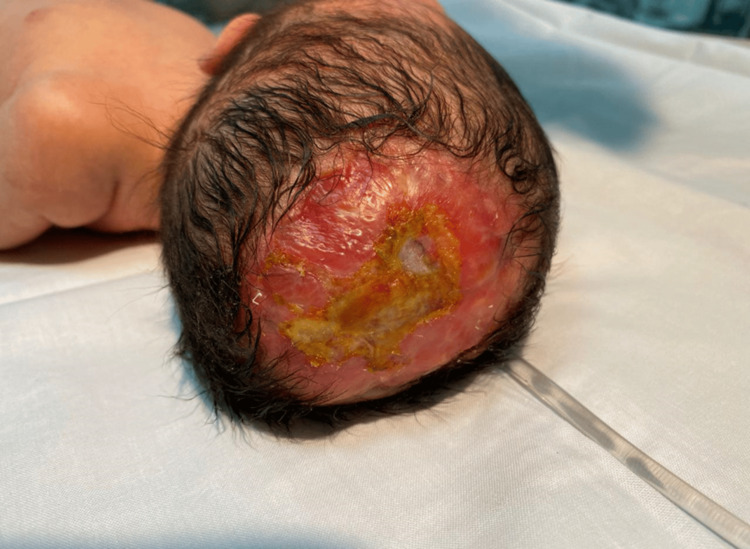
Skin aplasia of the vault with underlying bone defect.

On physical examination, the patient appeared healthy, without orofacial anomalies or evident dysmorphic features. A well-demarcated area of ACC, measuring approximately 10 × 5 cm, was observed in the midline parieto-occipital region of the scalp (Figure 1). The lesion presented as a scarred, atrophic plaque with extensive epidermal and dermal loss, exposing the underlying subcutaneous tissue and overlying a palpable cranial bone defect. The wound surface appeared erythematous and moist, with areas of fibrinous yellow exudate, suggestive of granulation tissue and early epithelialization. No active bleeding or purulent discharge was evident.

**Figure 2:**
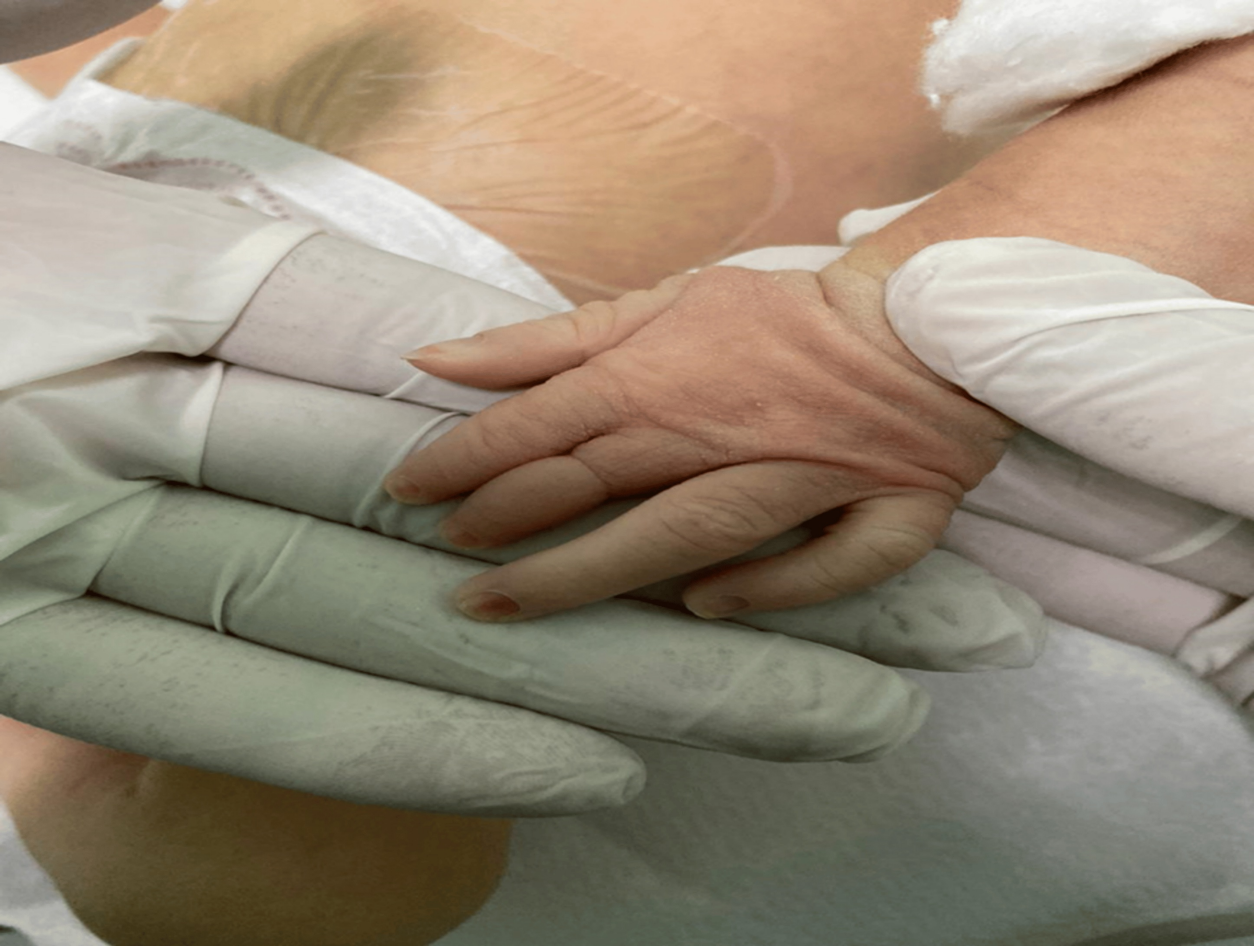
Brachydactyly and constriction band of the middle finger on the left hand.

Additionally, examination of the limbs revealed brachydactyly of the middle finger on the left hand (Figure 2), consistent with the terminal limb anomalies typically associated with AOS. A well-defined constriction band was also noted around the same digit. No other constriction bands were observed on the remaining limbs.

**Figure 3:**
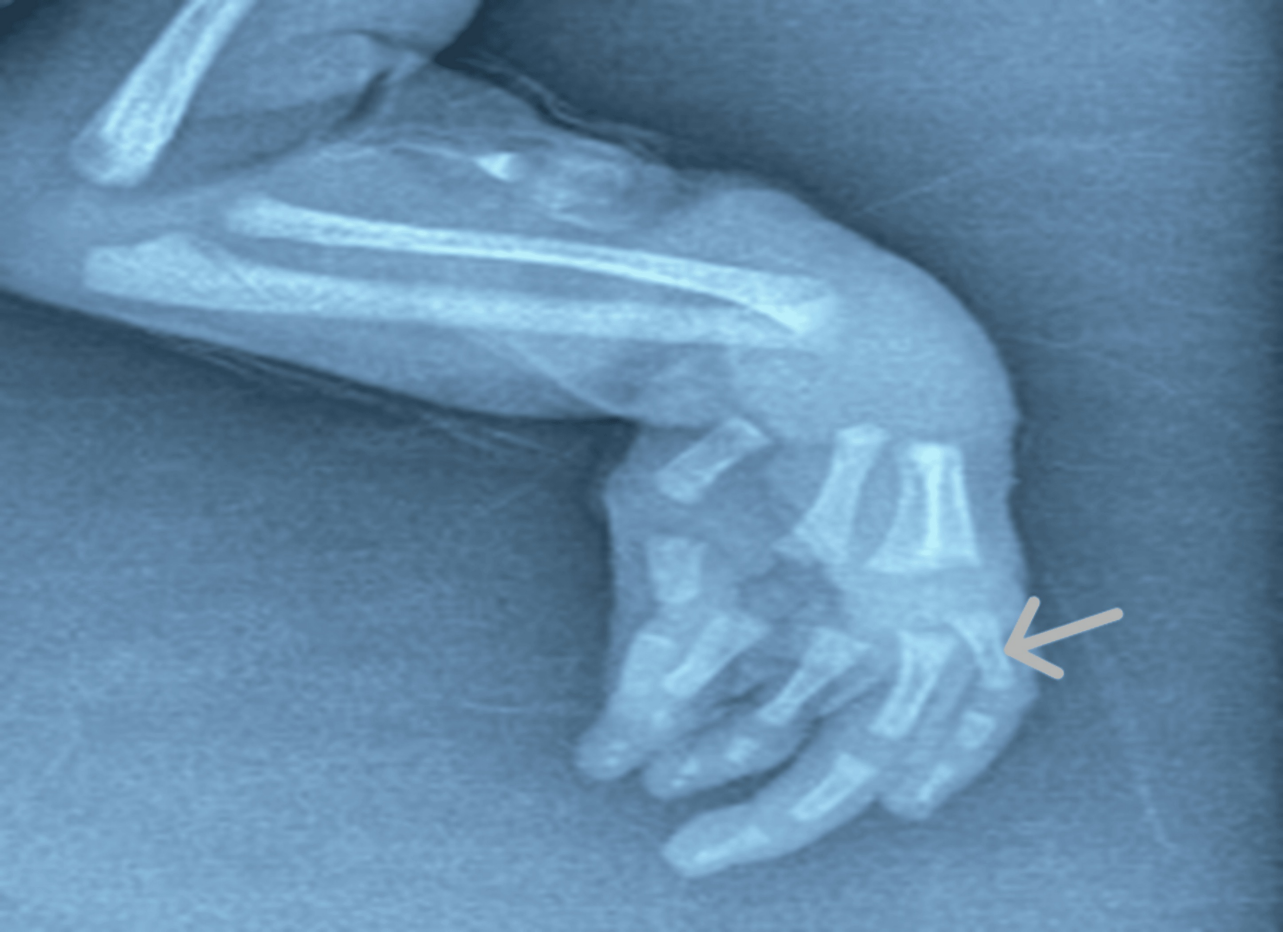
Radiograph of the left hand showing a shortened proximal phalanx of the middle finger (arrow).

**Figure 4:**
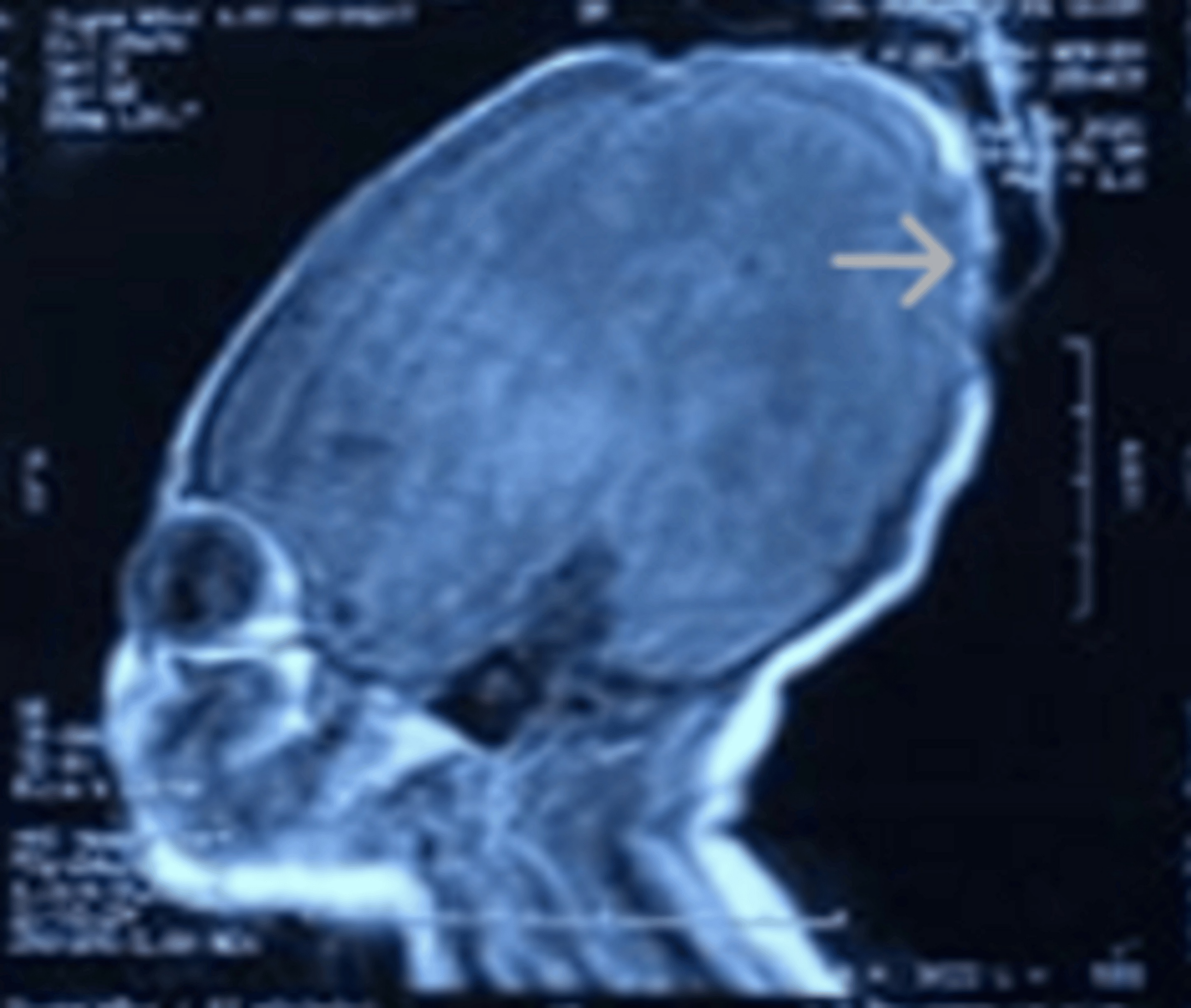
Cranial MRI of the patient demonstrating lack of ossification of the upper cranial vault (arrow).

A thorough clinical evaluation revealed no significant abnormalities across major organ systems. Cardiovascular, respiratory, gastrointestinal, renal, and neurological examinations were within normal limits. Abdominal and transthoracic ultrasonography demonstrated no structural anomalies, and ophthalmologic assessment was unremarkable. The patient exhibited normal spontaneous limb movements, with appropriate neonatal reflexes, including Moro, palmar grasp, and suckling. Plain radiographs of the left hand (Figure 3) revealed a shortened proximal phalanx of the middle finger, consistent with terminal limb anomalies commonly associated with AOS. Cranial magnetic resonance imaging (MRI) (Figure 4) demonstrated a lack of ossification of the upper cranial vault, while intracranial structures appeared normal, with no evidence of underlying brain malformations or other central nervous system abnormalities.

Initial management of the neonate’s ulcerated, hemorrhagic scalp lesion involved the application of a sterile fatty occlusive dressing to prevent secondary infection and minimize tissue maceration while awaiting surgical intervention. Intravenous antibiotics were administered, and nutrition was provided using maternal breast milk. On day two, alopecia of the vertex was corrected via scalp tissue expansion. The postoperative recovery was uneventful. Regarding the limb anomaly, no surgical treatment was pursued due to preserved hand function.

Genetic counseling was subsequently provided to the family to discuss the nature of AOS, its inheritance patterns, and implications for future pregnancies.

## Discussion

AOS is a rare hereditary disorder with an estimated incidence of approximately one in 225,000 live births [1,4]. It was first described in 1945 by Adams and Oliver, who reported eight affected individuals within a single family presenting with ACC of the scalp associated with transverse limb defects [1].

AOS demonstrates genetic heterogeneity with autosomal dominant (AD), autosomal recessive (AR), and sporadic cases reported [4]. Six genes have been identified: ARHGAP31, DLL4, NOTCH1, and RBPJ are linked to the AD form, while DOCK6 and EOGT correspond to the AR subtype [5]. Although AD inheritance with variable expression is most common, sporadic and AR cases also occur, the latter often associated with consanguinity [6].

In our case, the absence of affected family members suggests a sporadic occurrence, but consanguinity raises the possibility of an AR inheritance pattern. Thus, AOS may present sporadically or be inherited via AD or AR transmission, reflecting its complex genetic background.

The diagnosis of AOS is based on clinical criteria, divided into major and minor categories. Major criteria include terminal transverse limb defects, ACC, and a positive family history [4]. Minor criteria consist of cutis marmorata, congenital heart defects, and vascular anomalies. A diagnosis is established with either two major criteria or one major plus one minor criterion [4].

The clinical spectrum of AOS is highly variable, extending from asymptomatic presentations to severe, sometimes lethal forms associated with systemic complications [7]. Limb anomalies are the most frequent clinical manifestation of AOS, occurring in approximately 85% of cases [6]. These defects are typically asymmetric and more often affect the lower limbs. Reported anomalies include brachydactyly, syndactyly, polydactyly, oligodactyly, and nail hypoplasia, with severe cases showing complete absence of digits or limbs. Constriction rings and pseudo-amputations may also be present [7,8].

ACC is the second most common feature of AOS, seen in about 75-85% of patients [9]. It mainly affects the scalp, especially the parietal and vertex areas, with lesions typically measuring between 0.5 and 10 cm [7]. Less often, ACC can involve the trunk or limbs. In around 64% of cases with scalp involvement, there is an underlying skull defect, which may increase the risk during delivery, especially if instruments like forceps or vacuum are used [9]. Sometimes, skull defects occur without skin lesions and may be mistaken for a large fontanelle.

Approximately 20-25% of patients with AOS exhibit cutis marmorata telangiectasia congenita (CMTC), characterized by a persistent marbled skin pattern due to dilated superficial vessels. The condition can be localized or diffuse and may improve over time. Skin thinning and ulcerations can also occur [10]. Our patient did not show signs of CMTC.

Central nervous system involvement is reported in approximately 30% of AOS cases and may significantly affect prognosis [9]. Documented anomalies include microcephaly, cortical dysplasia, polymicrogyria, ventriculomegaly, hydrocephalus with periventricular and subthalamic calcifications, cerebellar and pontine hypoplasia, agenesis of the corpus callosum, and encephalocele [3]. These findings, increasingly recognized with advances in neuroimaging, may present alongside developmental delay or seizures. A more severe neurological phenotype with associated ocular anomalies has also been suggested [10].

Cardiac anomalies are reported in 23% of AOS cases and may contribute significantly to disease severity [9]. The most frequently described anomalies include left-sided obstructive defects (such as aortic coarctation and hypoplastic ventricles), septal defects, mitral and aortic valve malformations, and complex ones like tetralogy of Fallot [9,10]. Pulmonary complications, including arterial hypertension and venous stenosis, have also been documented [10]. In our case, no cardiac malformation was detected on echocardiography.

A wide range of less frequent anomalies has been reported in AOS, gastrointestinal, hepatic, genitourinary, ophthalmological (e.g., microphthalmia), skeletal, and craniofacial anomalies such as cleft lip [3,9]. None of these abnormalities were identified in our patient upon clinical evaluation and investigations.

## Conclusions

AOS is an uncommon, clinically heterogeneous, and potentially life-threatening congenital disorder in which internal organ systems are affected. However, in milder forms, as illustrated by our patient, the prognosis is favorable with limited cutaneous and digital involvement and no systemic anomalies. Surgical correction of scalp defects can yield good aesthetic outcomes. Regular follow-up remains essential for the early detection of potential complications. A multidisciplinary approach and genetic counseling are key to optimal management and future risk assessment.
